# Medical Schools

**Published:** 1841-09

**Authors:** 


MEDICAL SCHOOLS.

The Medical Department of the *University of New York* is at length fully organized, and its circular is before the profession.

DRS. HALL and PROUT of the *St. Louis Medical School* have resigned their professorships; and Dr. W. CARR LANE of that city, and Dr. RICHARD F. BARRETT of Springfield, Illinois, have been appointed their successors.

Dr. JNO. P. HARRISON, late Professor of Materia Medica in the Cincinnati College, has been appointed to the same chair in the *Medical College of Ohio*. D.

